# Coherent Control of a Few-Channel Hole Type Gatemon Qubit

**DOI:** 10.1021/acs.nanolett.4c00770

**Published:** 2024-06-07

**Authors:** Han Zheng, Luk Yi Cheung, Nikunj Sangwan, Artem Kononov, Roy Haller, Joost Ridderbos, Carlo Ciaccia, Jann Hinnerk Ungerer, Ang Li, Erik P.A.M. Bakkers, Andreas Baumgartner, Christian Schönenberger

**Affiliations:** † Quantum- and Nanoelectronics Lab, Department of Physics, University of Basel, 4056 Basel, Switzerland; ‡ MESA+ Institute for Nanotechnology University of Twente, 7500 AE Enschede, The Netherlands; § Department of Applied Physics, Eindhoven University of Technology, 5600 MB Eindhoven, The Netherlands; ∥ Swiss Nanoscience Institute, University of Basel, 4056 Basel, Switzerland

**Keywords:** superconducting qubit, nanowire, Josephson junction, germanium

## Abstract

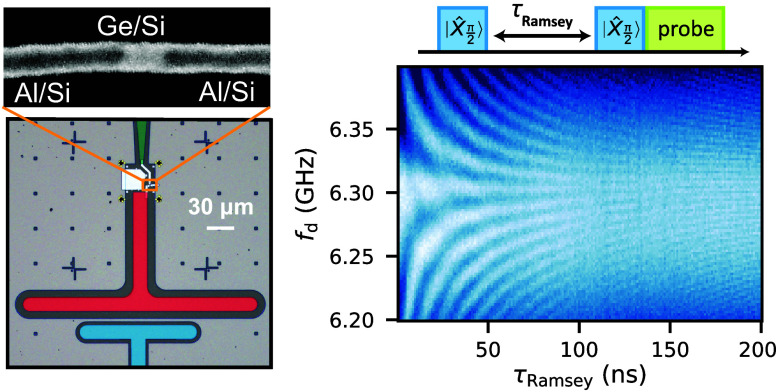

Gatemon qubits are the electrically tunable cousins of superconducting transmon qubits. In this work, we demonstrate the full coherent control of a gatemon qubit based on hole carriers in a Ge/Si core/shell nanowire, with the longest coherence times in group IV material gatemons to date. The key to these results is a high-quality Josephson junction obtained using a straightforward and reproducible annealing technique. We demonstrate that the transport through the narrow junction is dominated by only two quantum channels, with transparencies up to unity. This novel qubit platform holds great promise for quantum information applications, not only because it incorporates technologically relevant materials, but also because it provides new opportunities, like an ultrastrong spin–orbit coupling in the few-channel regime of Josephson junctions.

Quantum computing has become a topic of intense activity due to its potential to revolutionize information processing.^1^ Presently, one of the most popular platforms are superconducting circuits like transmon qubits, which are already applied to solve noisy intermediate-scale quantum problems.^2−4^ Conventional transmons rely on metallic superconductor–insulator-superconductor (SIS) tunnel junctions, in which the qubit frequency can only be tuned by a magnetic flux in a SQUID geometry.^5^ However, flux cross-talk between qubits and the heat load due to the flux control currents are problematic for scaling up the number of qubits.^6^

An alternative to metallic transmons are semiconductor-superconductor (Sm–S) hybrid systems,^7^ so-called gatemon qubits,^8,9^ with a wealth of recently demonstrated related concepts, like parity protected qubits,^10^ gate-tunable fluxonium qubits,^11^ gatemons operated with only a few highly transparent quantum channels,^12,13^ or Andreev level^14−16^ and Andreev spin qubits.^17−20^ All these devices rely on high-quality crystals with near-perfect Sm–S interfaces. This has limited the material choice mainly to InAs-based systems, like vertically grown InAs nanowires (NWs),^8,9,13,21,22^ InAs 2D systems,^23^ or selective-area-grown InAs NWs.^24^ However, III/V materials are difficult to integrate in standard CMOS technologies, and hyperfine interactions introduce additional decoherence.

More suitable for CMOS technology would be group IV materials, but only few gatemon-related experiments have been reported so far: on carbon-nanotubes,^25^ graphene,^26^ and large-diameter Ge/Si core/shell NWs,^27^ with qubit coherence times more than an order of magnitude shorter than in III/V materials. However, improvements are expected, especially for the technologically relevant CMOS compatible Si and Ge systems.^28,29^ In particular, Ge is of increasing interest for quantum information processing,^30^ because hole states in Ge have a p-wave symmetry, inherently reducing hyperfine interactions, with further improvements expected from isotopic purification. Even more promising might be Ge/Si core/shell nanowires with a 1D hole gas strongly confined by the Ge/Si interface^31,32^ and an electrically tunable, very large “direct” Rashba spin–orbit interaction.^33,34^

In this work, we report the full functionality of a hole-type gatemon qubit based on narrow Ge/Si core/shell NWs. The key step is the fabrication of highly transparent Josephson junctions (JJs), in an undemanding ex-situ annealing step^35,36^ driving a thermally activated propagation of superconducting aluminum into the Ge NW core.^37−40^ We incorporate such JJs in a gatemon qubit device and explicitly demonstrate the electrical tunability of the qubit frequency and the full coherent control of the qubit in the time domain, with an energy relaxation time on par with III/V systems. Most importantly, these experiments allow us to analyze the qubit anharmonicity, suggesting that the supercurrent through the junction is dominated by two essentially ballistic conductance channels with large transmissions to the reservoirs. This work establishes narrow Ge/Si core/shell NWs as a promising Sm–S hybrid platform for quantum information processing, and opens new avenues to investigate novel effects, for example, in circuit-quantum-electrodynamics (circuit QED) experiments on NWs^41,42^ with ultrastrong spin–orbit interactions.

Our gatemon qubit can be understood as a nonlinear LC oscillator that consists of a gate-tunable Ge/Si core/shell NW JJ as the nonlinear inductance, depicted in Figure 1a, and a shunt capacitor *C*_qb_ shown in Figure 1b. The NW has a diameter of ∼20 nm, and is expected to have minimal strain-induced defects and a high carrier mobility due to the [110] growth direction.^32^ Such a NW was transferred to an undoped Si/SiO_2_ substrate using a micromanipulator, and contacted by Al using standard lift-off techniques. The crucial step in forming the highly transparent JJ is a thermal annealing step at 200 °C, which drives Al atoms from the contacts into the Ge core, yielding an Al–Ge–Al junction with an atomically sharp interface.^39^ The Si shell remains intact during this process. The bright NW segment in the electron micrograph of Figure 1a shows the Ge core, while the Al-filled segments result in a darker contrast. The length of the Ge segment can be controlled by the annealing time, and directly read off as 33 nm for this specific device. The interface between Al and Ge turns out to be highly transparent, allowing for coherent transport of Cooper pairs through the semiconducting Ge.^37,43^ The Josephson energy *E*_J_ of this JJ can be tuned by a side gate voltage *V*_g_.

**Figure 1:**
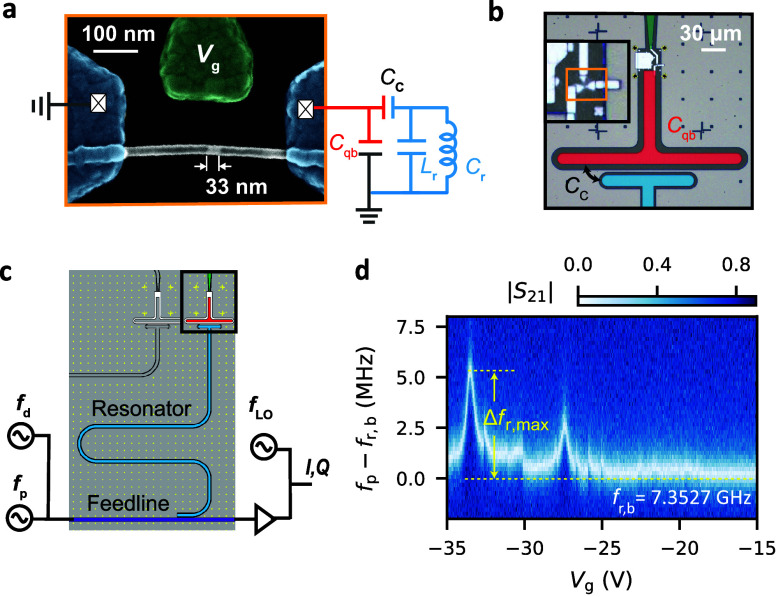
Ge/Si core/shell nanowire based gatemon device. (a) False-color scanning electron microscopy image of the Al–Ge–Al junction. The bright segment of the NW has a Ge core, while the dark segments contain Al. The side gate for electrical qubit control is colored green. The readout resonator is shown as a simplified equivalent circuit on the right. (b) False-color optical micrograph of a NW Josephson junction shunted by a T-shaped capacitor (red) to the surrounding ground plane and capacitively coupled (*C*_c_) to a superconducting λ/4 resonator (blue). (c) Overview of the circuit QED chip and schematic of the readout and control circuit. The λ/4 resonator is inductively coupled to a feedline for readout. (d) Transmission amplitude |*S*_21_| through the feedline as a function of the gate voltage *V*_g_ and the probe frequency *f*_p_, plotted as the difference from the bare resonator frequency *f*_r,b_. A bright signal signifies the resonator frequency *f*_r_.

The gatemon shunt capacitance *C*_qb_ (red) is provided by a T-shaped NbTiN island etched into the surrounding ground plane, as shown in Figure 1b. From electromagnetic simulations, we estimate *C*_qb_ ≈ 78 fF. The island is galvanically connected to the JJ, forming the nonlinear LC-oscillator, with the lowest two oscillator states forming the qubit states |0⟩ and |1⟩. The corresponding qubit frequency is determined by *E*_J_ and the charging energy *E*_c_ = *e*^2^/2*C*_qb_ ≈ *h* · 248 MHz. *E*_c_ is fixed and designed much smaller than the typical Josephson energy, significantly reducing the sensitivity to charge noise.^44^ In addition, we deposited a 100 nm thick Al layer on the surrounding NbTiN ground plane as a quasiparticle trap (see Supporting Information).

An overview of the circuit QED chip is shown in Figure 1c. The qubit state is probed via a capacitively coupled λ/4 coplanar transmission line resonator. The short end of this microwave resonator with a current antinode is inductively coupled to the feedline, while the open end with a voltage antinode is capacitively coupled to the qubit island via the qubit-resonator coupling capacitance *C*_c_. This device was measured in a dilution refrigerator with a base temperature of 15 mK, with consistent results in three different cooldowns.

Readout and manipulation of the gatemon qubit is accomplished using standard circuit QED techniques. When the qubit frequency *f*_q_ is strongly detuned from the resonator frequency *f*_r_, the system is in the dispersive regime.^45^ The qubit then causes a shift Δ*f*_r_ of the resonator frequency from its bare value *f*_r,b_, where Δ*f*_r_ = *f*_r_ – *f*_r,b_ ≈ (*g*/2π)^2^/(*f*_r,b_ – *f*_q_) is determined by the qubit-resonator coupling strength *g*. Figure 1d shows a measurement of the low power transmission amplitude |*S*_21_| plotted as a function of the gate voltage *V*_g_ and the probe frequency *f*_p_, plotted as the difference from the bare resonator frequency *f*_r,b_. The minimum in the transmission signal occurs whenever the probe frequency *f*_p_ is resonant with *f*_r_.

The bare resonator frequency *f*_r,b_ = 7.3527 GHz can be directly found at gate voltages *V*_g_ > – 15 V, where the semiconducting part of the NW is close to depletion, so that the Josephson current is negligible and the resonator frequency exhibits no shift. The corresponding internal quality factor of *Q*_i_ ≈ 2.41 × 10^5^ is found by simultaneously fitting the resonator transmission amplitude and phase in the vicinity of *f*_r,b_.^46^ At lower gate voltages, the hole density in the Ge segment is increased, resulting in a larger Josephson current on the order of a few tens of nA through the JJ, with a correspondingly larger Josephson energy and an increased qubit frequency. The resonator frequency is Lamb-shifted to higher values accordingly, with a maximum of Δ*f*_r,max_ ≈ 6 MHz at *V*_g_ ≈ – 33 V, pointed out in Figure 1d. The dispersive shift is positive, suggesting a qubit frequency lower than the bare resonator frequency in this gate voltage range. Δ*f*_r_ does not increase monotonically with gate voltage, possibly due to resonances in the semiconducting NW section.^43^ We also find discontinuities in *f*_r_ at specific gate voltages, for example at *V*_g_ ≈ 30 V. These, we attribute to a switching of the qubit frequency *f*_q_ due to charge traps in the vicinity of the NW. We note that the resonator frequency shift remains highly reproducible, including the switching, within a gate voltage range of a few volts, but becomes less reproducible when subjected to a wider gate sweep, probably due to rearrangements of localized charges.^22^

To study the quantized energy levels of the device, we perform pulsed two-tone spectroscopy. We apply a 500 ns drive tone at the frequency *f*_d_ to the feedline, followed by a 1 μs probe tone at an optimized frequency of *f*_p_ ≈ *f*_r_ to probe the resonator. We note that we obtain similar results when driving via the side gate. The in-phase *I* and quadrature *Q* components of the transmitted probe tone are obtained using heterodyne detection techniques. We plot the normalized amplitude *A*/*A*_0_ of the complex signal, which depends on the state of the qubit. In order to resolve fine features, we plot the numerically calculated second derivative of *A*/*A*_0_ with respect to *f*_d_, as shown in Figure 2a (raw data in Supporting Information Figure S5). To avoid large charge rearrangement, we focus on the small gate voltage interval of ∼600 mV shown in Figure 2a, in which the qubit frequency can be tuned between 5.4 and 6.3 GHz, with a typical qubit-resonator coupling factor of *g* ≈ 47 MHz. We attribute the reproducible discontinuity in the qubit frequency at *V*_g_ ≈ – 30.45 V to a nearby charge trap. We also find several gate-voltage independent sharp resonances, indicated by the yellow arrows in Figure 2a, possibly caused by two-level fluctuators more remote from the gate, for example in the SiO_2_ substrate, or due to spurious modes in the electromagnetic environment.^47^

**Figure 2:**
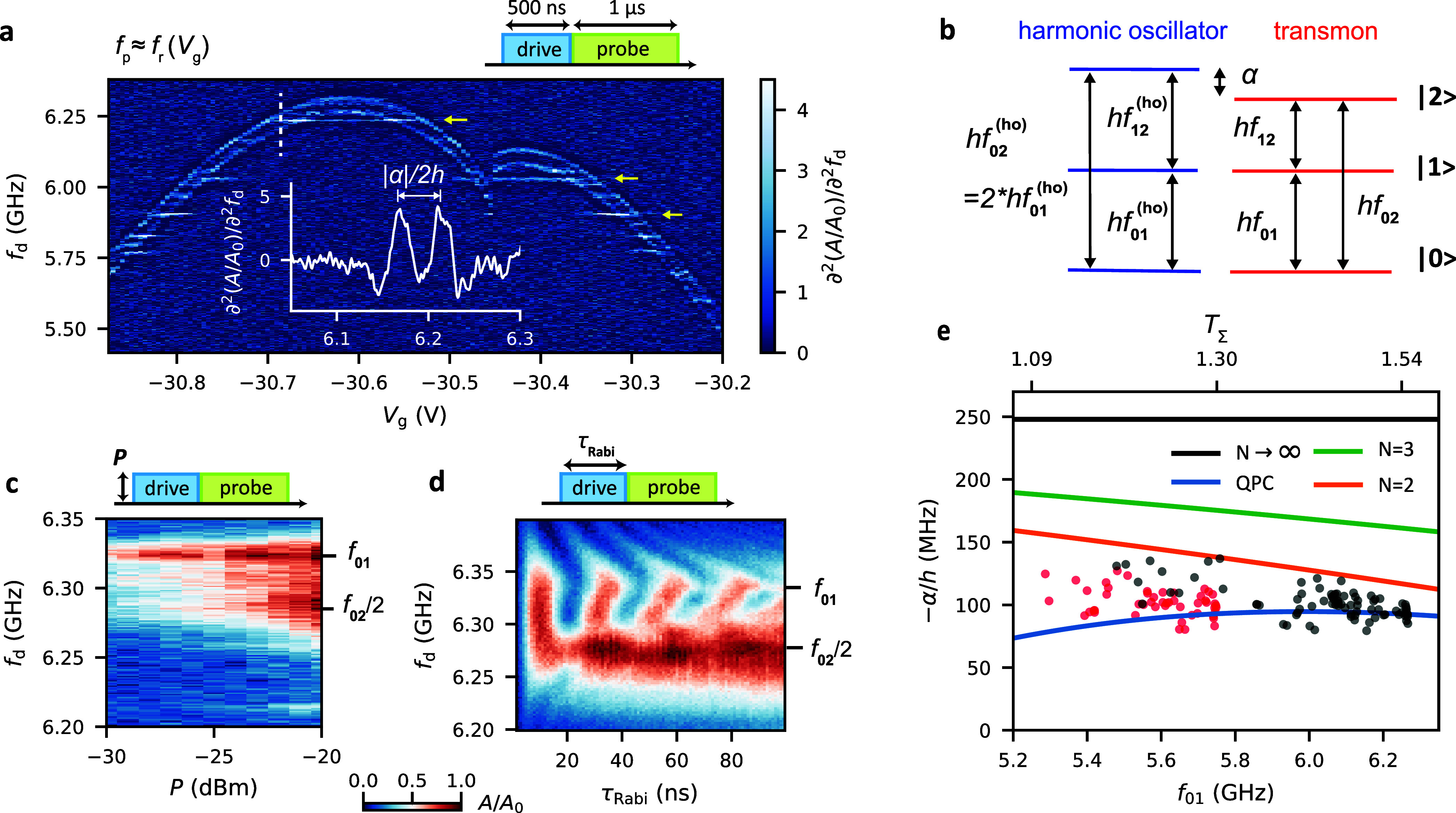
Qubit spectroscopy and anharmonicity. (a) Second derivative of the normalized resonator response *A*/*A*_0_ in a two-tone spectroscopy experiment as a function of the drive frequency *f*_d_ and the gate voltage *V*_g_. The pulse sequence is shown schematically on the top right. The drive power is set to *P* = −20 dBm and the probe frequency close to the resonator frequency *f*_r_. The inset shows a cross section at *V*_g_ = −30.7 V, clearly showing two peaks. (b) Energy diagram of a transmon qubit explicitly showing the anharmonicity α. (c,d) Normalized resonator response *A*/*A*_0_ in a two-tone spectroscopy experiment as a function of *f*_d_ and *P* and of *f*_d_ and the drive pulse duration τ_Rabi_, respectively. The latter exhibits characteristic Rabi oscillations. (e) Anharmonicity extracted from two-tone spectroscopy experiments, plotted as a function of the qubit frequency *f*_01_. The black data points were extracted from (a), while the red stem from larger gate voltages (around *V*_g_ ≈ −20 V). The top axis is the total transmission *T*_Σ_ obtained directly from the qubit frequency. The solid lines depict the anharmonicity inferred from the models discussed in the main text.

The main features in Figure 2a are two resonances, moving almost in parallel with changing *V*_g_, clearly visible in the cross section shown in the inset for *V*_g_ = −30.7 V. Here, the spacing between the two peaks is ∼50 MHz. Based on the generic energy diagram for a transmon qubit shown in Figure 2b, we attribute the higher frequency resonance to the qubit transition at frequency *f*_01_ between the ground state |0⟩ and the first excited state |1⟩, while the lower resonance originates from two-photon processes driving the transition from |0⟩ to the second excited state |2⟩.

To explicitly identify these two peaks, we perform two-tone spectroscopy as a function of frequency and drive tone power, as plotted in Figure 2c. At low powers, only a single resonance is found. For large powers, an additional resonance appears at a slightly lower frequency, consistent with a lower probability for higher order two-photon absorption processes. This transition to the second excited state is also found in the Rabi measurement shown in Figure 2d, where we plot the resonator response as a function of the drive pulse duration τ_Rabi_ and drive frequency *f*_d_. The drive pulse induces periodic Rabi oscillations between |0⟩ and |1⟩, resulting in the characteristic Rabi chevron pattern, with the qubit frequency *f*_01_ ≈ 6.325 GHz. Again, we find an additional broader feature for longer pulse times at ∼6.275 GHz, typically attributed to the *f*_02_/2 resonance.^26,48^ We point out that the Rabi experiments and the power dependence were not taken at the same gate voltage, because of a slight drift over several weeks of measurements.

This level structure now allows us to directly assess the anharmonicity of the gatemon spectrum α = 2*h*(*f*_02_/2 – *f*_01_), for different gate voltages. The corresponding data are plotted as black and red points as a function of the gate voltage dependent *f*_01_ in Figure 2e. We note that for some specific frequencies, spurious resonances hindered us from extracting the peak positions. Details about the data extraction are discussed in the Supporting Information.

Figure 2e is one of our main results, which we now use to estimate the transmission probability and the number of conducting channels in the NW junction. Since the semiconducting NW segment is very short (33 nm) compared to typical superconducting coherence lengths, the JJ is in the short-junction limit. The Josephson potential is then well described by , with Δ, *T*_*i*_, and ϕ̂ the superconducting gap, the individual channel transparencies, and the phase difference between the left and right Al segments.^49^ The gatemon Hamiltonian then reads , which can be expanded to fourth order in ϕ̂ around the potential minimum at ϕ̂ = 0, with the nonharmonic terms as perturbation to the harmonic oscillator solutions.^12^ This procedure yields
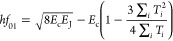
1and
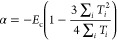
2with the Josephson energy given by the prefactor of the harmonic -term in the Hamiltonian, . Here, we use the gap Δ = 210 μeV found in DC transport experiments in a control device (see Supporting Information Figure S9). For this device, we estimate *E*_J_/*E*_c_ ≳ 80, so that we can approximate . Using the numerically simulated *E*_c_ and the measured qubit frequency *f*_01_, we obtain the total transmission , used as the top axis of Figure 2e. The measured quantities *hf*_01_ and α contain *T*_Σ_ and ∑_*i*_*T*_*i*_^2^, allowing us to estimate the number of channels and the corresponding transmission probabilities. To do this, we consider two limits.^12^ First, we assume *N* channels of equal transparency , yielding the anharmonicity . Figure 2e shows the cases *N* = 2, *N* = 3 and *N* → *∞*. This equal-transmission model provides an estimate of the number of active channels, with the case of *N* → *∞* giving the SIS tunnel junction limit α = −*E*_c_, with many low-transparency channels. All data points lie below the *N* = 2 case, suggesting that at most two channels dominate the JJ transport. The corresponding mean channel transparency is electrically tunable from T̅ ≈ 0.55 to T̅ ≈ 0.77 in this qubit frequency range.

In the second limit, often called the “quantum point contact (QPC) limit”, we assume one fully transmitting (*T*_I_ = 1) and one partially transmitting channel, with a free parameter *T*_II_. This limit yields the lowest possible |α| for a given *T*_Σ_. The resulting dependence is plotted in Figure 2e as blue solid curve. Since this curve captures our data better than the equal-transparency model, we conclude that the annealed Al–Ge–Al JJ carries one highly transparent channel, consistent with the large values up to *T* ≈ 0.96 reported in previous DC transport studies,^37,39^ and a second channel with a lower transmission, electrically tuned in the range from *T*_II_ ≈ 0.1 to *T*_II_ ≈ 0.54.

To demonstrate the full functionality of the gatemon qubit, we performed time-domain measurements, namely Rabi oscillations and Ramsey interferometry, and extract the corresponding relaxation and dephasing time. For these measurements, we use a Josephson parametric amplifier (JPA) to enhance the readout signal already at base temperature.^50^ In the following experiments, all pulse sequences start with a common initialization time of 100 μs to allow the qubit to relax to the ground state |0⟩. We plot the normalized resonator response *A*/*A*_0_, which essentially represents the occupation of state |1⟩. Each data point is averaged over 10000 identical pulse sequences.

First, we perform Rabi experiments at a fixed gate voltage *V*_g_ and drive at the frequency *f*_d_ ≈ *f*_01_. The drive pulse of duration τ_Rabi_ and power *P* is immediately followed by a 1 μs probe pulse. The drive pulse induces Rabi oscillations between the states |0⟩ and |1⟩ defining the *z*-axis of a Bloch sphere. The resulting qubit-state dependent resonator response is plotted in Figure 3a. As expected, the Rabi oscillations become faster for larger drive powers *P*, which allows for a calibration of the corresponding rotation on the Bloch sphere, around an axis we define as the *x*-axis. For example, a *X*_π/2_ pulse is obtained as a 5 ns drive pulse at *P* = −10 dBm. We note that we perform these experiments at low powers. If the qubit is driven at larger powers, the two-photon processes become more probable, resulting in a deviation from the linear dependence of the Rabi frequency on the drive amplitude for *hf*_Rabi_ ≳ α, and a corresponding leakage from the computational subspace^5^ (see Supporting Information Figure S6).

**Figure 3:**
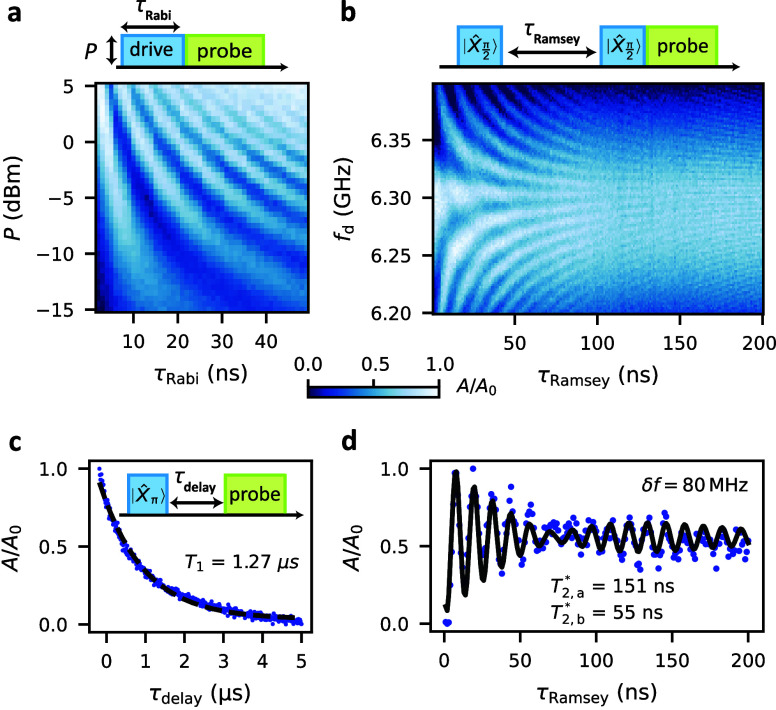
Coherent manipulation of the gatemon. (a) Rabi oscillations as a function of the drive power *P* and pulse duration τ_Rabi_, measured at *f*_d_ = 6.345 GHz and *V*_g_ = −30.7 V. (b) Ramsey fringes as a function of drive frequency *f*_d_ and time delay *t*_Ramsey_ measured at *V*_g_ = −30.6 V. Two *X*_π/2_ pulses separated by τ_Ramsey_ are applied prior to the probe tone. (c) Measurement of the characteristic energy relaxation time *T*_1_ at *V*_g_ = −30.7 V. A 10 ns *X*_π_ pulse at *P* = −10 dBm drives the qubit into state |1⟩. After a delay time τ_delay_, the qubit state is read out. The black dashed line shows a fit of an exponential decay, yielding *T*_1_ = 1.27 μs. (d) Ramsey experiment measured at *V*_g_ = −30.6 V at a detuning of 80 MHz. Two dephasing times of *T*_2,a_^*^ and *T*_2,b_^*^ are extracted by fitting to double sinusoidal functions with individual exponential envelopes.

Next, we perform Ramsey interferometry, with the corresponding pulse sequence shown schematically in the upper panel of Figure 3b. The qubit state vector is first rotated into the xy-plane of the Bloch sphere using a calibrated *X*_π/2_ pulse with the drive frequency *f*_d_ sightly detuned from the qubit frequency by *δf* = *f*_d_ – *f*_01_. During a subsequent delay time τ_Ramsey_, the qubit state precesses around the *z*-axis of the Bloch sphere, and a phase ϕ = 2*πδfτ*_Ramsey_ is accumulated. Then the state vector is again rotated by a π/2 pulse and the qubit occupation read out as a function of ϕ. This results in the Ramsey fringes shown in Figure 3b, where the resonator response is plotted as a function of *f*_d_ and τ_Ramsey_. The careful timing of these two types of pulses allows one in principle to reach any quantum superposition state on the Bloch sphere.

To quantitatively assess the qubit quality, we now measure the energy relaxation time *T*_1_ and the dephasing time *T*_2_^*^. First, to measure *T*_1_, a *X*_π_ pulse is applied to bring the qubit to state |1⟩. After a delay time τ_delay_, the occupation is measured with a probe pulse. Due to the qubit relaxing to the ground state, the probability of finding the qubit in the excited state decays exponentially over a characteristic time scale of *T*_1_ = 1.27 μs. Similarly, we extract the dephasing time *T*_2_^*^ from a Ramsey measurement at the detuning *δf* = 80 MHz, see Figure 3d. Instead of the expected single frequency with a dephasing time *T*_2_^*^, we find a beating pattern with two slightly different frequencies, similar to the one found in InAs NW gatemons^22^ and granular aluminum fluxonium qubits.^51^ From a fit to two sinusoidal functions with individual exponential decay times, we obtain the two frequency components *f*_Ramsey,a_ = 80.17 MHz and *f*_Ramsey,b_ = 86.05 MHz. The corresponding time constants are *T*_2,a_^*^ = 151 ns and *T*_2,b_^*^ = 55 ns. These two frequencies near *f*_01_ are also found in the high resolution drive power dependent two tone experiments shown in Supporting Information Figure S7. We tentatively attribute the two frequencies to two qubit configurations determined by a nearby low-frequency two-level fluctuator beyond our experimental control. Further qubit characteristics, a discussion of possible physical mechanisms limiting our qubit performance, and a comparison to other gatemons can be found in the Supporting Information. As a result, our device exhibits significantly longer coherence times than previously reported group IV gatemon qubits, comparable to recent experiments on InAs platforms.

In summary, we have demonstrated a fully functional gatemon qubit based on a narrow Ge/Si core/shell nanowire, with a highly transmissive few-channel Josephson junction fabricated using a simple annealing technique, without resorting to sophisticated epitaxial growth techniques. From a detailed analysis of the qubit anharmonicity, we conclude that the JJ is dominated by two quantum channels with transmissions up to unity. To demonstrate coherent control in the time domain, we performed Rabi and Ramsey interferometry experiments, yielding a *T*_1_ time on par with gatemons in the more established III/V materials. Since we find *T*_2_^*^ ≪ 2*T*_1_, the qubit coherence is not limited by the energy relaxation, but rather by dephasing caused by on-chip noise sources,^47,52^ suggesting that these times can still be improved significantly.

Our experiments show that Ge/Si core/shell NW gatemons are a competitive platform for electrically tunable supercondcuting qubits. JJs in the few-channel QPC limit might significantly reduce the charge noise sensitivity,^53−56^ possibly alleviating the requirement for large-footprint capacitors. Ge/Si core/shell NWs also offer additional design parameters not considered for superconducting qubits so far, namely the exceptionally strong and electrically tunable spin–orbit interaction of the hole carriers, and the tunable Landé *g*-factor.^33,34,57^ In addition, our material system and fabrication techniques open up new avenues to study Andreev bound states and other, more exotic subgap states, and can be used to fabricate other types of qubits, like Andreev spin qubits,^17−20^ or Andreev pair qubits,^14−16^ that will benefit from the reduced hyperfine interaction and the sharp interfaces to the Ge islands.

## Data Availability

All data in this publication are available in numerical form at: 10.5281/zenodo.10198946

## Supporting Information Available

Details of the fabrication methods, a device overview, two-tone spectroscopy with different drive powers, peak extraction procedure, power dependence of the Rabi oscillations, beating pattern in the Ramsey experiments, discussion of relaxation and dephasing mechanisms, superconducting gap measurement, resonator quality factor, and measurement setup. (PDF)
