# Supplementary material for: Coherent Control of a Few-Channel Hole Type Gatemon Qubit
Source: Nano Lett. 2024 Jun 7;24(24):7173–9. doi: 10.1021/acs.nanolett.4c00770 (PMC11194827; doi:10.1021/acs.nanolett.4c00770)
Supplement: Supplementary file 1 — nl4c00770_si_001.pdf [file nl4c00770_si_001.pdf]

Supplementary Information: Coherent control of a few-channel hole type gatemon qubit

Han Zheng,[1,*] Luk Yi Cheung,[1] Nikunj Sangwan,[1] Artem Kononov,[1] Roy Haller,[1] Joost Ridderbos,[2] Carlo Ciaccia,[1] Jann Hinnerk Ungerer,[1] Ang Li,[3] Erik P.A.M. Bakkers,[3] Andreas Baumgartner,[1,4,*] and Christian Schönenberger[1,4,*]

[1]*Quantum- and Nanoelectronics Lab, Department of Physics, University of Basel, 4056 Basel, Switzerland*

[2]*MESA+ Institute for Nanotechnology University of Twente, 7500 AE Enschede, The Netherlands*

[3]*Department of Applied Physics, Eindhoven University of Technology, 5600 MB Eindhoven, The Netherlands*

[4]*Swiss Nanoscience Institute, University of Basel, 4056 Basel, Switzerland*

(Dated: May 3, 2024)

I. DEVICE OVERVIEW

An optical micrograph of the circuit QED chip is shown in Fig. S1. Four qubits are in the center of the image, capacitively coupled to individual readout resonators (not fully shown). A 100 nm thick Al layer (bright square) was deposited on the NbTiN ground plane (gray background) as quasiparticle trap. Near the borders of the chip, we deposited several Au islands for better thermalization of the chip.

II. FABRICATION METHODS

The circuit QED chip was fabricated on an undoped silicon substrate with a 100 nm top thermal oxide, using a combination of optical and electron-beam lithography. After cleaning the wafer, a 68 nm NbTiN film was sputter deposited on the wafer, in which the resonator, feed line and gate line were patterned using optical lithography, and further defined using dry etching in an ICP-RIE process with Ar and Cl_2 gas. Next, we fabricated gold markers to later align the nanowires (NWs), and we deposited gold islands (size $\sim 500\,\mu\text{m} \cdot 500\,\mu\text{m}$) near the borders of the NbTiN film for better thermalization. The resulting resonator quality

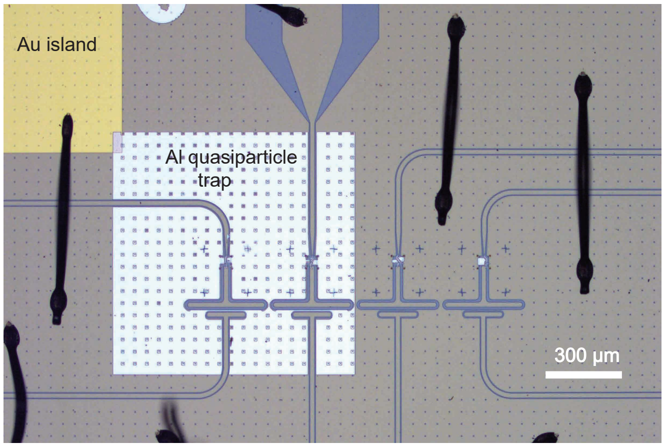

FIG. S1. Optical micrograph of the circuit QED chip. Four qubits were fabricated on each chip (center), coupled to the readout resonator (not fully shown). Al was deposited on the NbTiN ground plane as a quasiparticle trap and Au islands for better thermalization.

* Corresponding author:
han.zheng@unibas.ch;
andreas.baumgartner@unibas.ch;
christian.schoenenberger@unibas.ch

factors were obtained from fits shown in SI Fig. S2.

Ge/Si core/shell NWs were then transferred to the circuit QED chip using a micro-manipulator. Source and drain contacts as well as side gates were fabricated using standard electron beam lithography. To remove the native oxide on the Si shell, an 8 s wet etch in buffered HF (buffered oxide etchant 10:1, 4.6% HF) was performed, followed by rinsing in DI water. The chip was then immediately loaded into the evaporator where 40 nm of Al was deposited by thermal evaporation. After lift-off, the chip is annealed on a hotplate in air at 200°C for 10 min. The NWs were imaged in a scanning electron microscope to confirm the channel length. Importantly, the annealing and imaging steps can be iterated to obtain the targeted channel length. For each qubit island, we fabricated 3 or 4 annealed junctions, from which we selected the most promising and connected it to the capacitor and the grounding plane. Ar milling was used to remove the native Al oxide before contacting. The DC and RF wiring of the dilution refrigerator, as well as the measurement setups are discussed in SI Fig. S3.

III. RESONATOR QUALITY FACTOR

We use the Python based package "resonator_tools" [1] to extract the quality factors of our readout resonator. The data and fits of the magnitude and phase of the transmission signal S_{21} are shown in Fig. S2, yielding the internal and external quality factors.

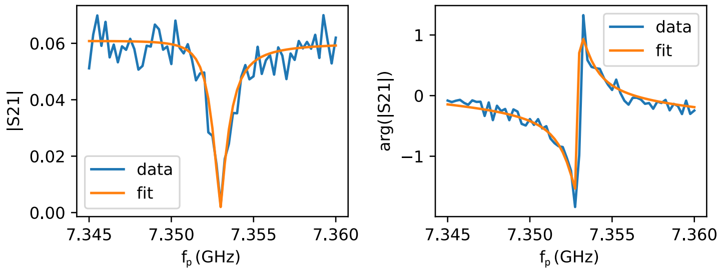

FIG. S2. The measured magnitude and phase of S_{21} in the vicinity of $f_{r,b}$. A simultaneous fit to both quantities using the model in Ref. [1] results in the internal quality factor $Q_\mathrm{i} \approx 2.41 \times 10^5$ and the external quality factor $Q_\mathrm{c} \approx 4 \times 10^3$.

IV. MEASUREMENT SETUP

The RF measurements were performed either using a Rohde&Schwarz ZNB-8 vector network analyzer or a Zurich Instrument SHFQA Quantum Analyzer. The drive and probe signals were heavily attenuated by 66 dB and filtered with home-made Ecosorb filters at low temperature. The output signals were amplified in an amplification chain consisting of a Josephson parametric amplifier (JPA), several circulators, an Ecosorb filter and dual junction isolators, followed by a HEMT amplifier. All components are shown in detail in Fig. S3.

The qubit drive tone is generated by a vector signal generator (Agilent E8267D) modulated by an envelope signal from an arbitrary waveform generator (AWG, Tektronix 5014C). The DC flux and gate lines are filtered using Ag epoxy filters at the coldplate and a 3-stage LC-filter with the cutoff frequencies 80 MHz, 225 MHz and 400 MHz. The qubit drive via the gate is achieved by combining a DC and an RF line on the PCB with a RC bias tee using a 1 kΩ resistor and a 15 nF capacitor.

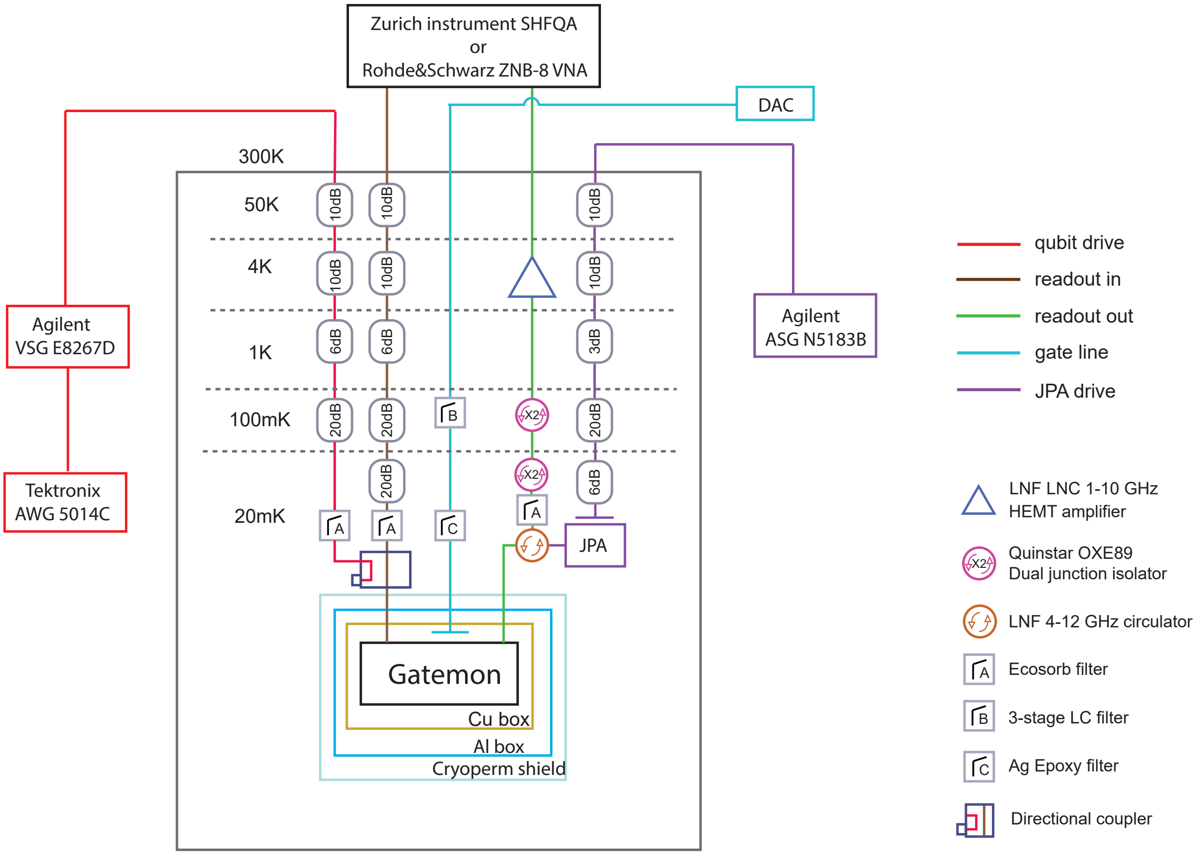

FIG. S3. Schematic of the dilution refrigerator and measurement setup.

V. TWO-TONE SPECTROSCOPY AT DIFFERENT DRIVE POWERS

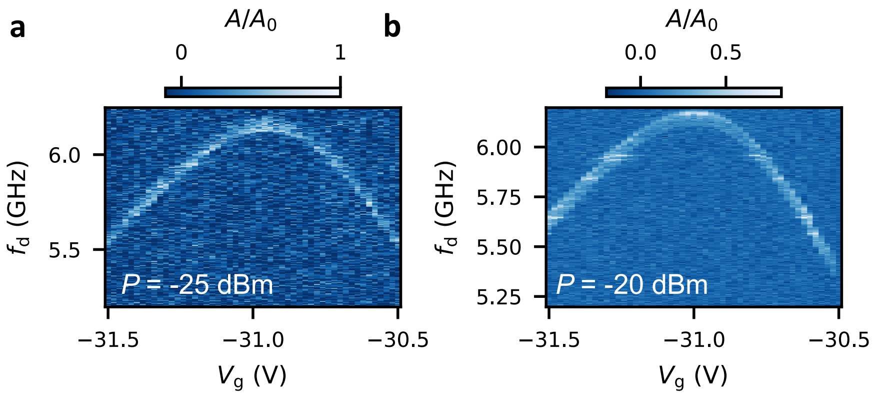

FIG. S4. Two-tone spectroscopy at different drive powers (a) P=-25 dbm and (b) P=-20 dbm. At low drive power, P=-25 dbm, only one peak is visible. At an elevated power P=-20 dbm, the second peak from the two-photon process can be resolved.

VI. PEAK EXTRACTION

In this section, we elucidate how we extracted the peak positions in the two-tone experiments. The raw data of Fig2,a is shown in Fig. S5a, with a cross section at $V_g = -30.7\,\mathrm{V}$ in the bottom panel. To better identify the double-peak structure already visible in the raw data, we first applied a moving average with a window size of 10 pixels along the frequency axis, and then took the second derivative. The frequency step size in the raw data is 1 MHz. A cross section of the processed data is displayed alongside the raw data in the lower panel of Fig. S5a.

Next, we extracted the peak positions as the positions of the maxima in the second derivative of the smoothed raw data. These positions are pointed out by red dots in the two-tone spectroscopy map in Fig. S5b. We manually discard peaks close to horizontal resonances that we attribute to gate voltage independent two-level fluctuators. From the difference between the two maxima we extract the anharmonicity, as discussed in the main text. Fig. S5c shows additional data and the corresponding second derivative for a different gate voltage range.

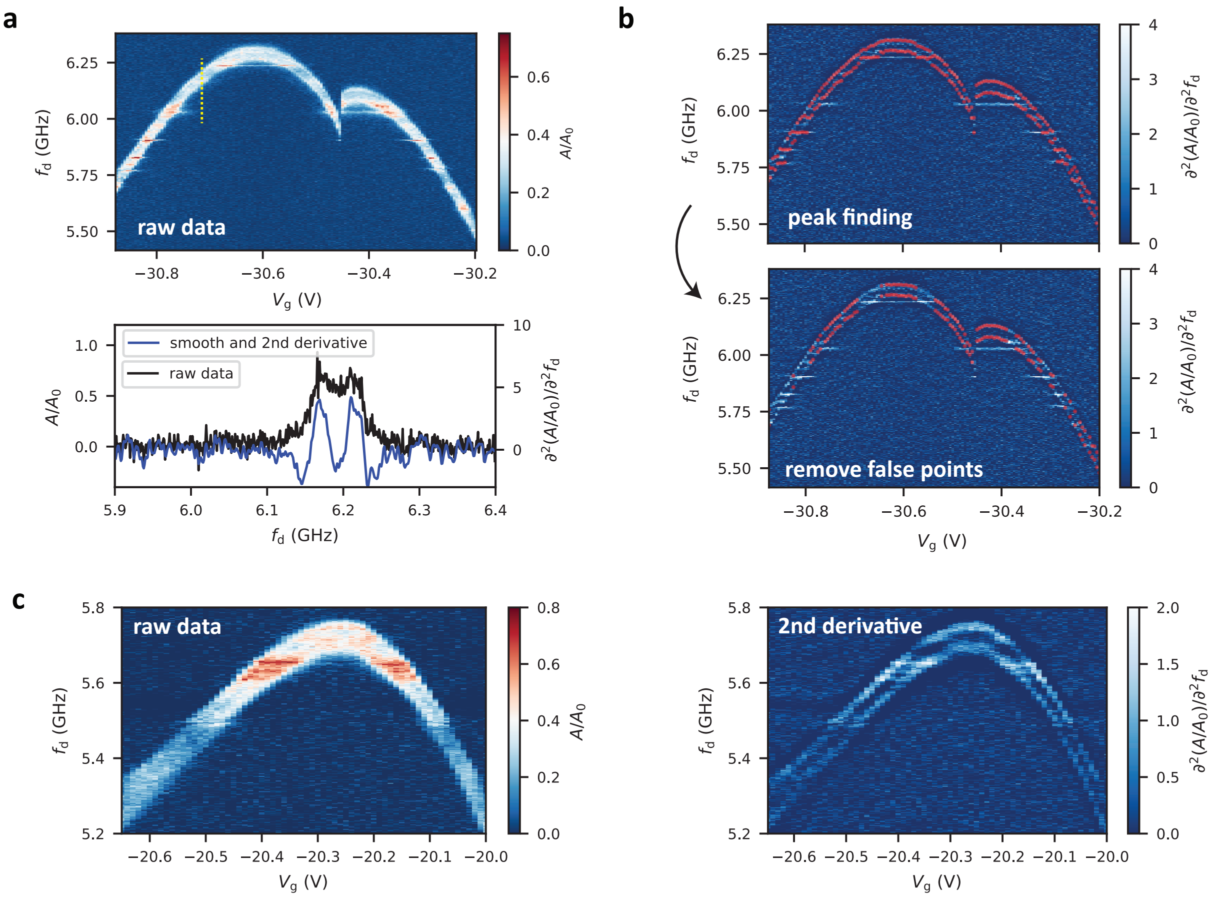

FIG. S5. **Raw data and peak extraction.** **a**, Raw data from Fig. 2a of the main text, showing the two-tone spectroscopy of the gatemon as a function of the drive frequency f_d and the gate voltage V_g. The indicated cross section (yellow dotted line) of raw data and the corresponding processed data are shown in the bottom panel. **b**, Extracted peak positions (red dots) superimposed on the two-tone spectroscopy map. Due to gate-independent spurious resonances, a subset of the peaks was disregarded. Only the ones shown in the bottom panel were used. **c**, Two-tone spectroscopy in a different gate voltage range used for the additional points in the anharmonicity plot of Fig. 2e of the main text. Left panel: raw data, right panel: second derivative of smoothed data.

VII. POWER DEPENDENCE OF THE RABI OSCILLATIONS

In a pure two-level system, the Rabi oscillations are faster for larger power, with a frequency proportional to the drive amplitude, or to the square root of the power. In Fig. S6a, we plot three examples of Rabi oscillations for the indicated (constant) powers. We then extract the Rabi frequency f_Rabi by fitting the data points to a sinusoidal function with an

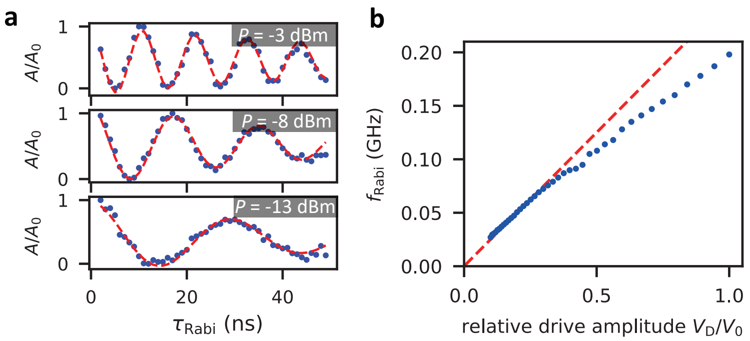

FIG. S6. **Power-dependent Rabi measurements.** **a**, Rabi oscillations at different drive powers $P = -3\,$dBm, $P = -8\,$dBm and $P = -13\,$dBm, as indicated. A sinusoidal function with exponential decay (dashed red line) is fitted to the measured data (blue points) to extract the Rabi frequency f_{Rabi} **b**, f_{Rabi} as a function of the relative drive amplitude V_{D}/V_0. The red dashed line is a linear fit in the low power range.

exponentially decaying envelope. The resulting f_{Rabi} is plotted as a function of the relative drive amplitude $V_{\text{d}}/V_0 = \sqrt{P/P_0}$ in Fig. S6b. For low amplitudes, f_{Rabi} increases linearly with V_{d}, as expected. However, for f_{Rabi} larger than $\sim 80\,$MHz, the observed frequency clearly deviates from the low-amplitude linear dependence. This deviation can be directly attributed to transitions to the second excited state of the gatemon, or in other words, to a leaking out of the computational sub-space. This effect can have two physical origins: 1) a larger power results in a larger occupation of the first excited state and therefore in a larger probability of the two-photon processes that drive the $|0\rangle \rightarrow |2\rangle$ transition. 2) drive pulses of short duration result in a broader frequency spectrum, if assuming a Gaussian broadened pulse shape [2], starting to drive the $|1\rangle \rightarrow |2\rangle$ transition [2]. The deviation from the low-power linear dependence occurs around $\sim 80\,$MHz, consistent with the anharmonicity found in the main text. This discussion directly illustrates how the anharmonicity limits the qubit operation speed.

VIII. BEATING PATTERN IN RAMSEY EXPERIMENTS

In Fig. S7, a high-resolution power-dependent two-tone spectroscopy experiment is presented. In addition to the resonances at f_{01} and $f_{02}/2$ discussed in the main text, a third transition at a frequency offset δf above f_{01} is visible, with δf ranging from $5\,$MHz to $10\,$MHz. We tentatively attribute this resonance to a power dependent fast switching be-

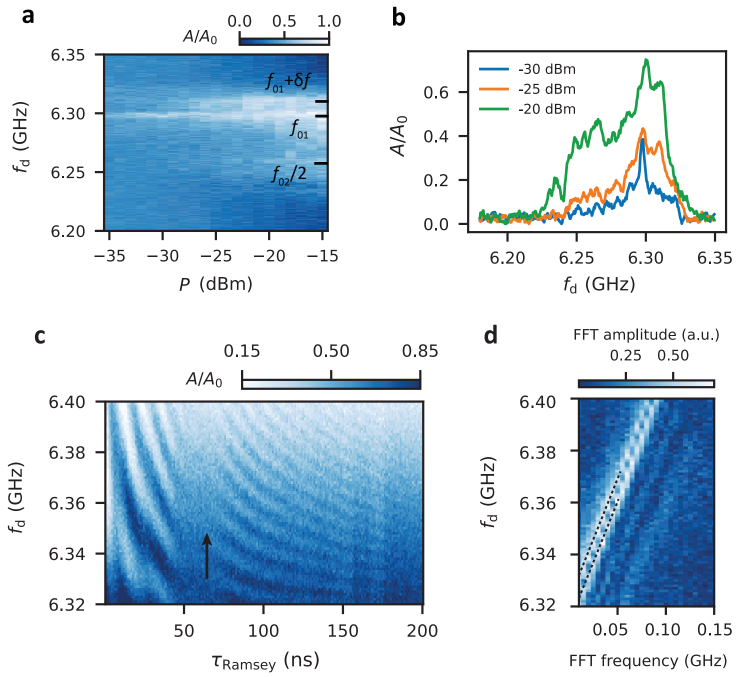

FIG. S7. **Beating pattern in Ramsey measurement.** **a**, Two-tone spectroscopy as a function of drive power P and drive frequency f_d. Specific cross sections at different drive powers are shown in **b**. **c**, Example of a Ramsey type measurement described in the main text. The signal vanishes intermittently around $\tau_\mathrm{Ramsey} \sim 70\,\mathrm{ns}$, pointed out by a black arrow, as discussed in the main text. **d**, Fast Fourier transform of **c** showing two main qubit frequencies, indicated by black dashed lines.

tween two slightly different values of f_{01}, which leads to the beating pattern observed in our Ramsey measurements. Figure S7c shows an example in which the beating is clearly visible. The signal vanishes around a delay time of $\tau_\mathrm{Ramsey} \sim 70\,\mathrm{ns}$, and re-appears for higher values. The Fourier transform of these data is shown in Fig. S7d, in which two different frequency components are found close to the main qubit transition f_{01} (dashed lines).

The switching between two qubit frequencies could have various origins. In our case, two possibilities can be excluded: 1) quasiparticle poisoning [3, 4] would block one or more transport channels and therefore change the Josephson energy, and with it the qubit frequency. Since there are only two channels dominating the transport in our JJ (see main text), a poisoning would change the qubit frequency by several GHz, not merely by a few ten MHz as found in our experiments. 2) fluctuations in the Cooper pair number [5] should occur on the kHz, not on the MHz scale, based on the ratio $E_\mathrm{J}/E_\mathrm{c} \approx 80$. After these considerations, We tentatively attribute the switching frequency to a nearby bi-stable charge fluctuator [6].

IX. RELAXATION AND DEPHASING MECHANISMS

With changes in the gate voltage (and therefore the qubit frequency), T_1 changes seemingly randomly between 0.6 to 1.3 μs. Similar to [7–9], we cannot find a clear correlation between T_1 and f_{01} or df_{01}/dV_g. Moreover, the obtained T_1 values are much lower than $T_{\text{Purcell}} = 1/(2\pi\kappa(g/\Delta^2)) \approx 100\mu$s ($\kappa = 1.5$ MHz and $\Delta = 1.5$ GHZ), suggesting that the qubit relaxation time is not limited by the Purcell decay [10] into the readout resonator. We tentatively attribute the gate dependence of T_1 to two-level fluctuators weakly coupled to the qubit, resulting in various energy-relaxation channels [11]. This is supported by the finding that after leaving the device in ambient conditions for several weeks, the average T_1 dropped by a factor of ~ 2. This suggests that with more impurities adsorbed on the nanowire surface, the qubit energy relaxation time T_1 drops considerably.

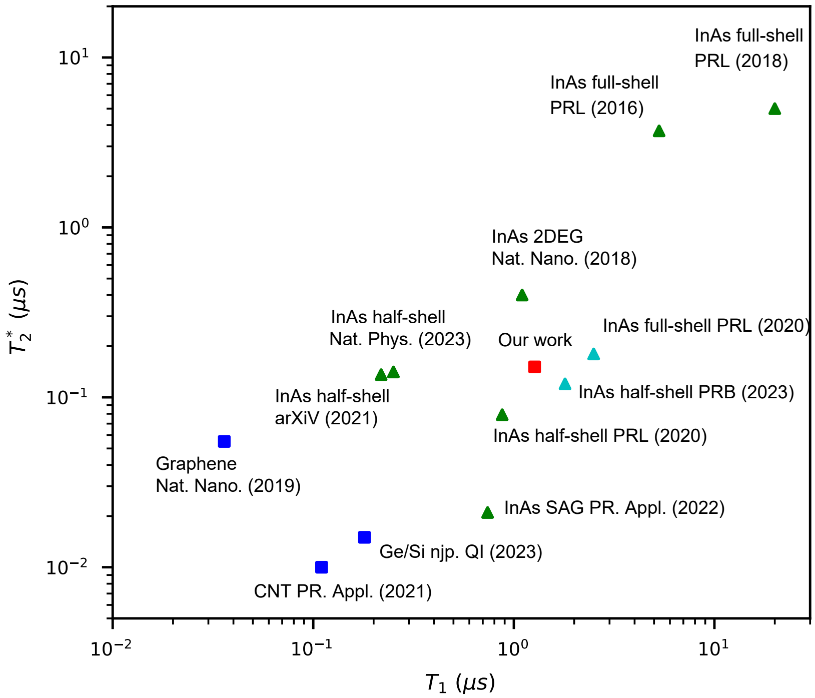

FIG. S8. **Comaprison of transmon coherence times for different material platforms.** The plot shows literature values for T_1 and T_2^* times for various experiments in the literature. Squares symbolize group IV devices, green and cyan triangles stand for III/V InAs-based ones. The cyan data points is a subset of group III/V gatemon experiments in which T_2^* was not reported and we substituted it by T_{Rabi}. The data and the corresponding references are listed in Table S1

In our gatemon, $T_2^* \ll 2T_1$, showing that the qubit coherence is not limited by energy relaxation. In addition, we did not find a significant improvement of T_2^* on and off the gate

Material	Platform	Superconductor	$T_1 (\mu s)$	$T_2^* (\mu s)$	$T_{2,echo} (\mu s)$	# channels
III/V	InAs NW (full-shell)[12]	Epitaxy	5.3	3.7	9.5	2 to 3 [13]
III/V	InAs NW (full-shell) [6]	Epitaxy	20	5	30	2
III/V	InAs NW (full-shell) [14]	Epitaxy	0.251	0.141	0.33	
III/V	InAs NW (full-shell) [15]	Epitaxy	0.25	0.18		
III/V	InAs NW (half-shell) [16]	Epitaxy	1.8	0.12		
III/V	InAs NW (half-shell) [17]	Epitaxy	0.218		0.136	
III/V	InAs NW (half-shell) [18]	Epitaxy	0.875	0.079	0.295	
III/V	InAs 2DEG [8]	Epitaxy	1.1	0.4	2.2	many
III/V	InAs SAG NW [19]	Epitaxy	0.74	0.021	1.34	2
IV	Graphene [9]	Surface	0.036	0.055		many
IV	Carbon nanotube [20]	Surface	0.11	(0.01)		1
IV	Ge/Si core/shell thick NW [21]	Al-Ge exchange	0.18	(0.015)		many
IV	Ge/Si core/shell thin NW (our work)	Al-Ge exchange	1.27	0.151		2

TABLE S1. **Comparison of gatemon platforms** The columns in this table show the material (group III/V or group IV), the exact platform, the types of superconducting contacts, the best reported qubit characteristics times T_1, T_2^*, $T_{2,echo}$, as well as the number of channels active in the JJ. Numbers in brackets indicate that T_2^* was not directly measured in the Ramsey experiment, but deduced from the qubit linewidth. For data points in cyan color, T_2^* were not reported in the experiment, we therefore use T_{Rabi} instead.

sweet spots, for example at $V_\mathrm{g} \approx -30.6\,\mathrm{V}$, nor with better filtering on the DC gate-line by replacing the 80 MHz low-pass LC filters by 100 kHz RC filters. From these findings, we conclude that T_2^* is not limited by gate voltage noise, but rather by on-chip noise originating for example from localized states in the native oxide of the Si shell or in thermal oxide of the substrate, resist residues from fabrication, or from carbon contamination from SEM imaging.

To compare the coherence times of our gatemon to other platforms, we show the results from prior studies in Fig. S8 and in Table. S1. Our gatemon exhibits the best coherence times of the rather few group IV based platforms to date, on a similar scale as recent InAs based materials with epitaxial Al.

X. SUPERCONDUCTING GAP MEASUREMENT

The superconducting gap Δ is measured in a control device, shown in Fig. S9a. The device is fabricated with the same annealing method, with a Ge channel length of 250 nm. The device was cooled down in the same dilution refrigerator as the circuit QED chip.

When the NW is gate-tuned close to pinch off, the NW channel forms a quantum dot, tunnel coupled to the annealed Al in the NW. In this regime, we can perform tunnel spectroscopy to determine the superconducting gap Δ. For this, we measured the differential conductance $\partial I_{\mathrm{D}}/\partial V_{\mathrm{SD}}$ as a function of source-drain voltage bias V_{SD} and side gate voltage V_{SG}. As shown in Fig. S9b, we find Coulomb blockade diamonds with a gap of 2Δ in the transport opened around zero bias. A cross section at the indicated gate voltage is plotted in Fig. S9c, where we extract $\Delta \approx 210\,\mu$eV.

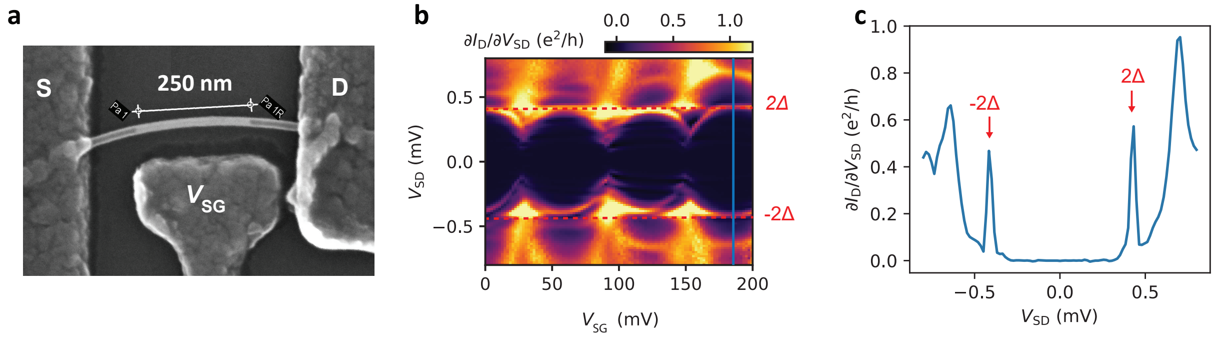

FIG. S9. **Superconducting gap from DC transport.** **a**, SEM image of a Ge/Si core/shell nanowire (NW) JJ fabricated with the same annealing method as in the main text. **b**, DC voltage bias spectroscopy as a function of the gate voltage V_{SG} and the source-drain bias voltage V_{SD}. A cross section at $V_{\mathrm{SG}} = 187\,\mathrm{mV}$ is shown in **c**.

---

[1] S. Probst, F. Song, P. A. Bushev, A. V. Ustinov, and M. Weides, Efficient and robust analysis of complex scattering data under noise in microwave resonators, Review of Scientific Instruments **86**, 024706 (2015).

[2] P. Krantz, M. Kjaergaard, F. Yan, T. P. Orlando, S. Gustavsson, and W. D. Oliver, A quantum engineer's guide to superconducting qubits, Applied physics reviews **6**, 021318 (2019).

[3] X. Pan, Y. Zhou, H. Yuan, L. Nie, W. Wei, L. Zhang, J. Li, S. Liu, Z. H. Jiang, G. Catelani,

et al., Engineering superconducting qubits to reduce quasiparticles and charge noise, Nature Communications **13**, 7196 (2022).

[4] J. Aumentado, G. Catelani, and K. Serniak, Quasiparticle poisoning in superconducting quantum computers, Physics Today **76**, 34 (2023).

[5] J. Koch, M. Y. Terri, J. Gambetta, A. A. Houck, D. I. Schuster, J. Majer, A. Blais, M. H. Devoret, S. M. Girvin, and R. J. Schoelkopf, Charge-insensitive qubit design derived from the cooper pair box, Physical Review A **76**, 042319 (2007).

[6] F. Luthi, T. Stavenga, O. Enzing, A. Bruno, C. Dickel, N. Langford, M. A. Rol, T. S. Jespersen, J. Nygård, P. Krogstrup, *et al.*, Evolution of nanowire transmon qubits and their coherence in a magnetic field, Physical review letters **120**, 100502 (2018).

[7] G. De Lange, B. Van Heck, A. Bruno, D. Van Woerkom, A. Geresdi, S. Plissard, E. Bakkers, A. Akhmerov, and L. DiCarlo, Realization of microwave quantum circuits using hybrid superconducting-semiconducting nanowire josephson elements, Physical review letters **115**, 127002 (2015).

[8] L. Casparis, M. R. Connolly, M. Kjaergaard, N. J. Pearson, A. Kringhøj, T. W. Larsen, F. Kuemmeth, T. Wang, C. Thomas, S. Gronin, *et al.*, Superconducting gatemon qubit based on a proximitized two-dimensional electron gas, Nature nanotechnology **13**, 915 (2018).

[9] J. I.-J. Wang, D. Rodan-Legrain, L. Bretheau, D. L. Campbell, B. Kannan, D. Kim, M. Kjaergaard, P. Krantz, G. O. Samach, F. Yan, *et al.*, Coherent control of a hybrid superconducting circuit made with graphene-based van der waals heterostructures, Nature nanotechnology **14**, 120 (2019).

[10] A. Houck, J. Schreier, B. Johnson, J. Chow, J. Koch, J. Gambetta, D. Schuster, L. Frunzio, M. Devoret, S. Girvin, *et al.*, Controlling the spontaneous emission of a superconducting transmon qubit, Physical review letters **101**, 080502 (2008).

[11] P. Klimov, J. Kelly, Z. Chen, M. Neeley, A. Megrant, B. Burkett, R. Barends, K. Arya, B. Chiaro, Y. Chen, *et al.*, Fluctuations of energy-relaxation times in superconducting qubits, Physical review letters **121**, 090502 (2018).

[12] L. Casparis, T. Larsen, M. Olsen, F. Kuemmeth, P. Krogstrup, J. Nygård, K. Petersson, and C. Marcus, Gatemon benchmarking and two-qubit operations, Physical review letters **116**, 150505 (2016).

[13] A. Kringhøj, L. Casparis, M. Hell, T. W. Larsen, F. Kuemmeth, M. Leijnse, K. Flensberg, P. Krogstrup, J. Nygård, K. D. Petersson, *et al.*, Anharmonicity of a superconducting qubit

with a few-mode josephson junction, Physical Review B **97**, 060508 (2018).

[14] M. Pita-Vidal, A. Bargerbos, R. Žitko, L. J. Splitthoff, L. Grünhaupt, J. J. Wesdorp, Y. Liu, L. P. Kouwenhoven, R. Aguado, B. van Heck, *et al.*, Direct manipulation of a superconducting spin qubit strongly coupled to a transmon qubit, Nature Physics **9**, 1110–1115 (2023).

[15] D. Sabonis, O. Erlandsson, A. Kringhøj, B. Van Heck, T. W. Larsen, I. Petkovic, P. Krogstrup, K. D. Petersson, and C. M. Marcus, Destructive little-parks effect in a full-shell nanowire-based transmon, Physical Review Letters **125**, 156804 (2020).

[16] A. Danilenko, D. Sabonis, G. W. Winkler, O. Erlandsson, P. Krogstrup, and C. M. Marcus, Few-mode to mesoscopic junctions in gatemon qubits, Physical Review B **108**, L020505 (2023).

[17] W. Uilhoorn, J. G. Kroll, A. Bargerbos, S. D. Nabi, C.-K. Yang, P. Krogstrup, L. P. Kouwenhoven, A. Kou, and G. de Lange, Quasiparticle trapping by orbital effect in a hybrid superconducting-semiconducting circuit, arXiv preprint arXiv:2105.11038 (2021).

[18] A. Bargerbos, W. Uilhoorn, C.-K. Yang, P. Krogstrup, L. P. Kouwenhoven, G. De Lange, B. Van Heck, and A. Kou, Observation of vanishing charge dispersion of a nearly open superconducting island, Physical review letters **124**, 246802 (2020).

[19] A. Hertel, M. Eichinger, L. O. Andersen, D. M. van Zanten, S. Kallatt, P. Scarlino, A. Kringhøj, J. M. Chavez-Garcia, G. C. Gardner, S. Gronin, *et al.*, Gate-tunable transmon using selective-area-grown superconductor-semiconductor hybrid structures on silicon, Physical Review Applied **18**, 034042 (2022).

[20] M. Mergenthaler, A. Nersisyan, A. Patterson, M. Esposito, A. Baumgartner, C. Schönenberger, G. A. D. Briggs, E. A. Laird, and P. J. Leek, Circuit quantum electrodynamics with carbon-nanotube-based superconducting quantum circuits, Physical Review Applied **15**, 064050 (2021).

[21] E. Zhuo, Z. Lyu, X. Sun, A. Li, B. Li, Z. Ji, J. Fan, E. Bakkers, X. Han, X. Song, *et al.*, Hole-type superconducting gatemon qubit based on ge/si core/shell nanowires, npj Quantum Information **9**, 51 (2023).
